# Synthesis and Electrochemical Study of Gold(I) Carbene Complexes

**DOI:** 10.3390/molecules29174081

**Published:** 2024-08-28

**Authors:** Andrea Rodríguez-Rubio, Álvaro Yuste, Tomás Torroba, Gabriel García-Herbosa, José V. Cuevas-Vicario

**Affiliations:** Departamento de Química, Facultad de Ciencias, Universidad de Burgos, 09001 Burgos, Spain; andrearr@ubu.es (A.R.-R.); ayusrod@itq.upv.es (Á.Y.); ttorroba@ubu.es (T.T.); gherbosa@ubu.es (G.G.-H.)

**Keywords:** Gold(I), NHC-complexes, electrochemistry, DFT calculations, Carbon Dioxide Reduction Reaction

## Abstract

In this work, we have prepared and characterized some gold compounds wearing a N-heterocyclic carbene (NHC) ligand as well as alkynyl derivatives with different substituents. The study of their electrochemical behavior reveals that these complexes show an irreversible wave at potentials ranging between −2.79 and −2.91 V, referenced to the ferrocenium/ferrocene pair. DFT calculations indicate that the reduction occurs mainly on the aryl−C≡C fragment. The cyclic voltammetry experiments under CO_2_ atmosphere show an increase in the faradaic current of the reduction wave compared to the experiments under argon atmosphere, indicating a possible catalytic activity towards the carbon dioxide reduction reaction (CO_2_RR).

## 1. Introduction

In recent years, gold(I) N-heterocyclic carbenes (NHCs) have emerged as interesting compounds with several applications [1,2]. Among these applications, several uses in bioinorganic applications have been recently reviewed [3,4,5]. Examples of these complexes behave as therapeutics for cancer treatment [6,7,8] or antibacterial agents [9]. In addition to these biological applications, gold(I) NHCs are involved as catalysts in a wide number of reactions [10,11]. In some recent works, the use of gold(I) NHCs as catalysts has been described for different reactions such as asymmetric catalysis [12,13,14], the synthesis of small rings [15], C–H bond activations [16,17], cross-coupling reactions [18], hydration reactions [19] and aqueous-phase catalysis [20], showing the high interest in gold complexes as catalysts for different reactions.

The carbon dioxide reduction reaction (CO_2_RR) is a very interesting possibility for transforming CO_2_ into valuable fuels and chemicals using renewable energy [21]. Several approaches such as biological [22,23,24,25], thermochemical [26,27,28], photochemical [29,30,31,32] and electrochemical [25,33,34,35,36,37,38,39,40,41] are under study to convert CO_2_ into formic acid, carbon monoxide, methanol, methane or other compounds with higher numbers of carbon atoms. Among these different approaches, electrochemical reduction has some advantages, such as the possibility of working at mild conditions of pressure and temperature, product selectivity by controlling the applied potential and the possibility of obtaining the necessary electricity from renewable sources [33,42]. The mild conditions used in the electrochemical reduction open up the possibility of using homogenous catalysis. Several examples of homogeneous electrocatalysis with transition metals have been reported [43,44,45].

In the context of the use of CO_2_ as a feedstock, several activation reactions of this molecule have been reported. In these reactions, gold(I) NHCs catalyze the carboxylation of activated C–H bonds [46,47], carboxylative cyclization [47,48,49] or the formation of a trigold carbonate complex from atmospheric CO_2_ [50]. This activation of the CO_2_ may encourage the search for gold complexes as possible catalysts of the CO_2_RR. Although gold is used in some instances as an electrode in this process, few examples involving organometallics or coordination compounds [51] or clusters [51,52,53,54,55,56,57,58] are reported. In all cases, the reaction product was CO, but a small amount of formic acid could be detected in the solution [53]. In the present work, we describe the synthesis and characterization of alkynyl-gold(I) complexes with N-heterocycle carbene ligands, and we explore their reactivity towards the electrocatalyzed CO_2_RR.

## 2. Results and Discussion

**Scheme 1:**
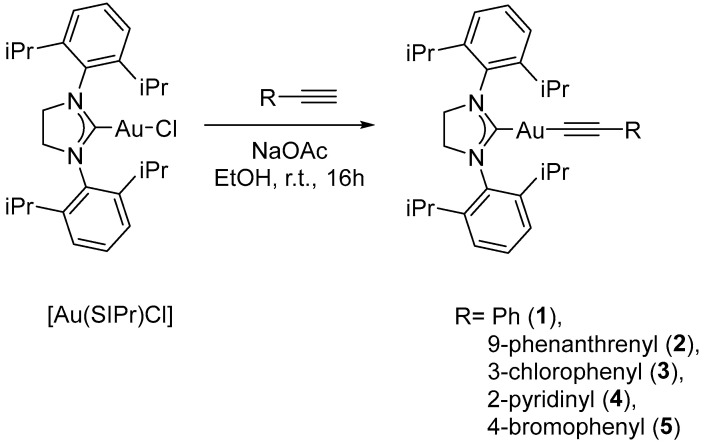
General procedure of synthesis.

The reaction of a gold complex [Au(SIPr)Cl] with different arylalkynes in the presence of sodium acetate following the procedure described by Nolan et al. [59] yielded the compounds **1**–**5** (Scheme 1).

These complexes were characterized by FT-IR, ^1^H NMR spectroscopies and elemental analysis. The infrared spectra of these compounds feature a characteristic band at ~2115 cm^−1^ for ῦ(C≡C). The NMR spectra in DMSO-d_6_ (Figures S1–S4) show a characteristic sharp singlet at 4.11–4.07 ppm that corresponds to the equivalent four nuclei of the hydrogen atoms of the methylene groups of the NHC ring. The isopropyl signals appear as a septuplet at 3.17–3.10 ppm and two doublets at 1.43–1.35 and 1.42–1.31 ppm. The aromatic protons from the aryl-alkyne and the diisopropyl-phenyl rings appear mixed in the 8.55–6.91 ppm interval.

### 2.1. X-ray Structure Analyses

**Figure 1:**
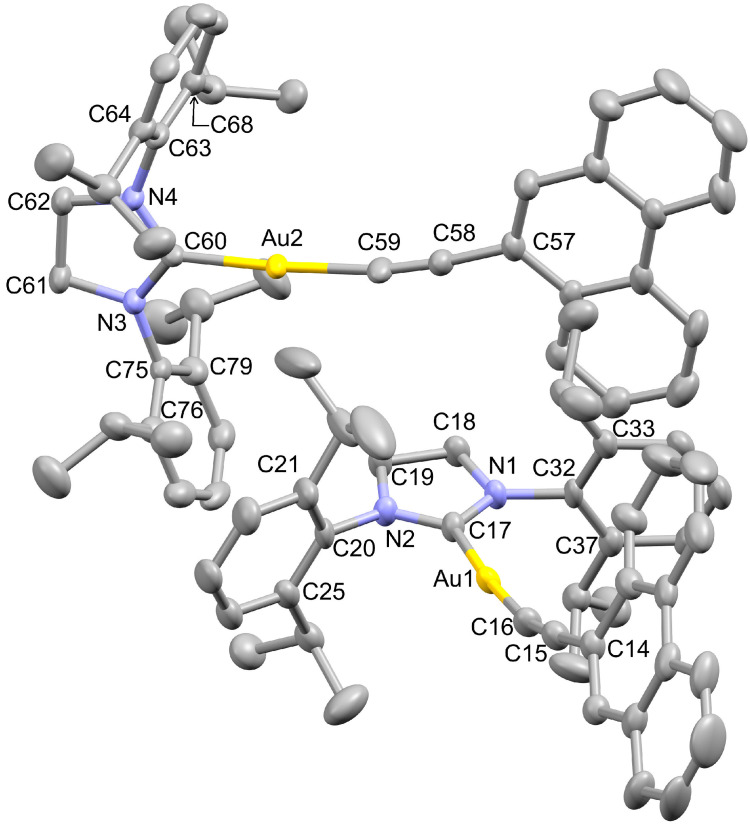
Molecular structure and atomic labeling of complex **2**. Hydrogen atoms are omitted to see the structures more clearly. Thermal ellipsoids are 30%. The code of colors of the atoms is: Carbon (grey), Nitrogen (blue), Gold (yellow).

Single crystals suitable for X-ray diffraction (XRD) analysis were obtained by slow diffusion of hexane over a concentrated solution of **2** in CHCl_3_. The X-ray molecular structure of this complex and its atom numbering are shown in Figure 1. Table 1 collects the crystallographic data, and Table 2 gathers some structural features of these complexes. This compound crystallized in the orthorhombic system with the *P*2_1_2_1_2_1_ space group. The asymmetric unit contains two molecules. In one of these molecules, the ring of the NHC and the plane of the phenanthrene form an angle of 10.15°, and in the other molecule, the angle involving the same fragments is 63.79°. The molecular structure found is characterized by the gold atom being two-coordinated by two carbon atoms in an almost linear arrangement with C–Au–C angles of 176.1 (3) and 173.8 (3). The couple of fragments interact with each other through two C–H···H–C interactions [60]. One of these interactions involves two methyl groups of different fragments, and the other interaction is established between a C−H of an isopropyl group of one fragment and a C−H of the phenanthrenyl group of the other fragment (see Figure S5). The fragments of the asymmetric unit interact with other analogous complexes of gold to build up the crystal. These interactions are C−H···H−C and C−H···π towards the aromatic rings and the triple C≡C bond (see Figures S6 and S7).

### 2.2. Electrochemical Measurements

**Figure 2:**
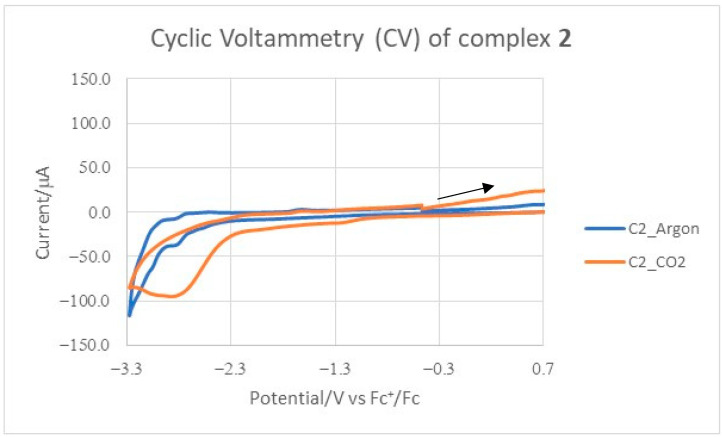
Fragment of cyclic voltammogram of complex **2** (5·10^−4^ M, glassy carbon working electrode dish with 3.0 mm diameter, dry MeCN, 0.1 M Bu_4_NPF_6_, scan rate 100 mV·s^−1^), recorded under argon atmosphere (blue line) and CO_2_ atmosphere (orange line). The arrow indicates the scan direction. The complete cyclic voltammogram can be seen in Figure S5 of the Supplementary Materials.

The electrochemistry of these complexes in acetonitrile solution was studied using cyclic voltammetry and Bu_4_NPF_6_ as a supporting electrolyte. The values of the cyclic voltammetry are collected in Table 3. In these measurements, a AgCl/Ag (3 M KCl) reference electrode was used, and at the end of the experiment, ferrocene was added as an internal calibrant. With the only exception being **1**, upon scanning to negative potentials under argon, all complexes display irreversible reduction waves at negative values of potential under argon atmosphere (blue line in Figure 2 and Figures S8–S12). Compounds **2**–**4** display an irreversible reduction wave between −2.87 and −2.91 V versus the ferrocenium/ferrocene (Fc^+^/Fc) couple. Compound **5** has an irreversible reduction at −2.79 V, and complex **1** shows no similar reduction wave. The irreversible characteristic of the reduction wave is related to the evolution of the reduction product. These values are close to the other reported values for carbene complexes of gold(I) with amido ligands [61,62]. Gold(I) carbene complexes with halogen atoms completing the coordination of the metal (instead of the alkynyl ligands reported in this work) usually show less negative values for the reduction wave ranging from −1.22 V to −1.82 V [63,64,65], although there is reported an example in which the carbene is a ferrocenyl-substituted 1,2,3-triazolylidene ligand, showing a reduction wave at −2.47 V [66]. In gold complexes, this wave is generally assigned to the reduction of Au(I) to Au(0) [67], but we found (see below, quantum chemical calculations and Table S1) that the topology of the lowest unoccupied molecular orbital (LUMO) of these complexes locates this molecular orbital mainly over the C≡C–Aryl fragments (a participation ranging between 81.40 and 91.03% of the orbitals of the C≡C–Aryl on the LUMO) with little participation of the atomic orbitals of the gold (ranging between 7.41 and 15.65%). As the LUMO of these complexes is mainly spread over the aryl–C≡C fragment, the reduction process occurs mainly in these fragments of the complexes, although with a small participation of the gold center. In the case of complex **1**, the high value of the energy of the LUMO can explain the lack of value for the reduction potential under our experimental conditions because this value should appear out of the window of measurement of the experiment [68,69,70,71].

Several NHC complexes of transition metals that are electrocatalysts of the CO_2_RR yielding CO have been reported. Re(I) with pyridyl–NHC or pyrimidyl−NHC chelating ligands are efficient catalysts in the reduction of CO_2_ to CO [72,73]. Related Mn(I) complexes with pyridyl–NHC ligands [74], and the same metal with chelating bis-NHC ligands [75,76], display electrocatalytic activity for the reduction of CO_2_ to CO. Fe(II) complexes with analogous pyridyl−NHC ligands or chelating bis-NHC and terpyridine as redox-active ligands also show electrocatalytic activity towards the CO_2_RR, yielding CO [77,78]. The combination of pyridyl–NHC and terpyridine ligands coordinated to Ru(II) generates electrocatalysts for the same reaction as the analogous combination of Fe(II) [79], but a similar complex immobilized on an N-doped porous carbon electrode reduced the CO_2_ to ethanol [80]. In addition to these complexes, bis-NHC ligands with different connectors such as pyridine (CNC pincer ligand) [81], xanthene (COC flexible and hemilabile ligand) [82] or methylene [83] show this electrocatalytic activity as well. Complexes of Co(II) [84] or Ni(II) [85] with macrocyclic ligands that were composed of a bipyridine fragment and two NHCs were active in this reaction. Pd(II) complexes with bis-NHC ligands bearing unique onium functionalities facilitate CO_2_ coordination to the catalytic center and enhance catalytic selectivity for the conversion of CO_2_ to CO compared to H_2_ production [86]. To the best of our knowledge, no gold NHC complexes with this activity have been reported. When the cyclic voltammetry experiments are performed under CO_2_ atmosphere, an increase in the cathodic current is observed (orange lines in Figure 2 and Figures S4−S8). This variation on the voltammogram is indicative of a CO_2_-electrocatalyzed reduction. The comparison of the electrochemical activity of the complexes can be analyzed from the values of the *i*_CO2_/*i*_Ar_ ratio. These values are ranging between 1.77 and 2.61. Figure 2 shows the results obtained for complex **2** as a representative example.

### 2.3. Quantum Chemical Calculations

**Figure 3:**
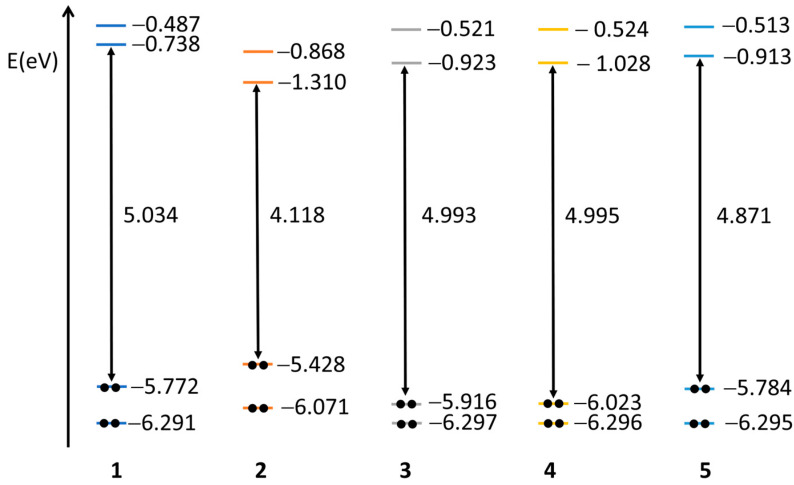
Calculated energy levels for the FMOs of complexes **1**–**5**.

**Figure 4:**
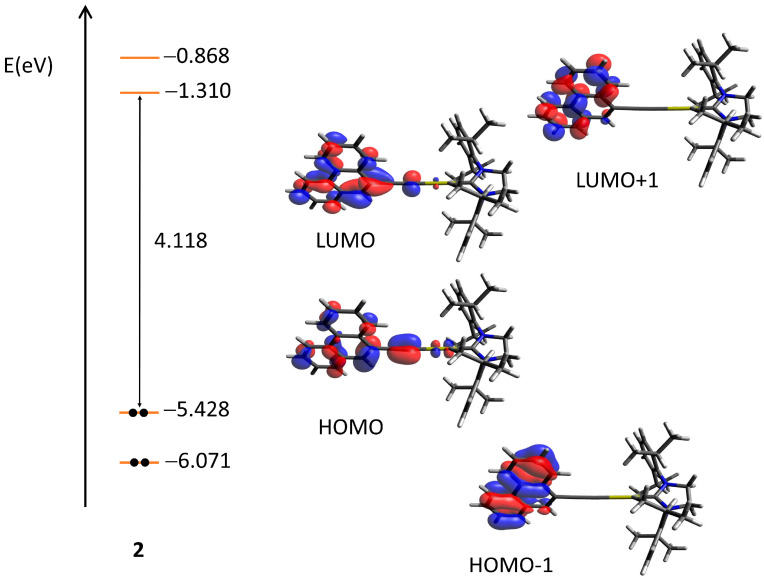
Energy levels and isosurface contour plots (0.03 au) for complex **2**.

DFT calculations were performed in order to obtain a better understanding of the electronic structure of these complexes (see Section 3 for details). Table 2 gathers some calculated structural values of the optimized structure for complex **2** in its ground state (S_0_), which showed only positive frequencies, and the comparison with the experimental XRD values, showing a good agreement between the structural parameters, validating the level of theory. Figure 3 shows the energy levels of the frontier molecular orbitals (FMOs) for all complexes. For complexes **1**–**5**, both the highest occupied molecular orbital (HOMO) and the LUMO are mainly located on the aryl-alkynyl fragment, with some small participation of the metal center, as can be seen in Figure 4 and Figures S9–S12 and Table S1. This topology of the LUMO indicates that the entry of the electron in the reduction process occurs mainly on this fragment. The high value of the energy of the LUMO in complex **1** correlates well with the high value of the reduction potential for complex **1** [68,69,70,71].

## 3. Materials and Methods

Unless otherwise stated, all reagents were purchased from commercial suppliers and used without further purification. Specifically, 2-ethynylpyridine, 1-bromo-4-ethynylbenzene and 3-Chloro-1-ethynylbenzene were purchased from Thermo Scientific (Waltham, MA, USA), 9-ethynylphenanthrene was purchased from TCI and HAuCl_4_·3H_2_O was purchased from Johnson Matthey (London, UK). Complexes [Au(NHC)(Cl)] and **1** were prepared following previously reported procedures [59]. A JASCO FT/IR-4200 spectrometer equipped with an ATR accessory model JASCO ATR PRO ONE was used for the recording of infrared spectra. Nuclear magnetic resonance (NMR) spectra were recorded with Varian Mercury-300 and BRUKER AVANCE III HD 300 MHz spectrometers. These measurements were performed at room temperature (25 °C) using DMSO-d_6_ as a solvent. The reference used for reporting the chemical shifts (*δ*) was the residual solvent peaks, and the units were parts per million (ppm), rounded to the nearest 0.01. For signals with coupling between nuclei, spin–spin coupling constants (*J*) were given in Hz, rounded to the nearest 0.1 Hz, and peak multiplicity was indicated as *s* (singlet), *d* (doublet), *t* (triplet), *q* (quartet), *sept* (septuplet), *m* (multiplet) and *br* (broad). The purity of the complexes was tested through elemental analyses that were performed with a Thermo Fisher Scientific EA Flash 2000 elemental microanalyzer.

### 3.1. Electrochemical Measurements

Cyclic voltammetry measurements were performed using portable potentiostat/galvanostat PalmSens3 (PalmSens, Houten, The Netherlands) equipment controlled by the software PSTrace4 Version 4.4.2. These experiments of cyclic voltammetry were carried out under argon or CO_2_ atmosphere. The cell had three electrodes; one of them was a glassy carbon disc (diameter = 3 mm) acting as the working electrode, another was a platinum wire acting as the auxiliary electrode and the third electrode was a Ag/AgCl (MF-2052 BASi) reference electrode separated from the bulk solution by a Vycor frit. At the beginning of the experiments, oxygen was removed from the solution by bubbling argon for 10 min and keeping the current of argon constant throughout the whole experiment. The solvent used in the solutions was MeCN, and the concentration was 5 × 10^−4^ M in all cases. [*n-*Bu_4_N][PF_6_] (0.1 M) was used as the supporting electrolyte. The scan rate of the cyclic voltammetry was 100 mV·s^–1^ in a clockwise direction. At the end of all the experiments, ferrocene was added as the internal reference. The potential experimentally determined for the redox couple Fc^+^/Fc was E_1/2_° = 0.476 ± 0.002 V vs. Ag/AgCl.

### 3.2. Quantum Chemical Calculations

DFT calculations were performed using Becke’s three-parameter B3LYP exchange–correlation functional [87,88] implemented in Gaussian16 [89]. The basis sets used to define the atoms were LANL2DZ [90] for Au and 6-31G (d,p) [91,92] for the other atoms. The empirical dispersion correction was taken into account using Grimme’s dispersion with Becke–Johnson damping, GD3BJ [93,94]. The solvent (MeCN) effects were considered within the self-consistent reaction field theory using the solvation model SMD, as described by Marenich et al [95].

### 3.3. X-ray Crystallography

A single crystal of **2** was mounted on a glass fiber and transferred to a Bruker X8 APEX II CCD-based diffractometer equipped with a graphite-monochromated Cu K_α_ radiation source (λ = 0.71073 Å). The highly redundant data sets were integrated using SAINT [96] and corrected for Lorentz and polarization effects. The absorption correction was based on the function fitting to the empirical transmission surface, as sampled by multiple equivalent measurements with the program SADABS [97]. The software package SHELXTL (version 6.10) [98] was used for space group determination, structure solution and refinement with full-matrix least-squares methods based on F2. A successful solution by direct methods provided most non-hydrogen atoms from the E map. The remaining non-hydrogen atoms were located in an alternating series of least-squares cycles and difference Fourier maps. All non-hydrogen atoms were refined with anisotropic displacement coefficients. Hydrogen atoms were placed using a “riding model” and included in the refinement at calculated positions. CCDC 2363526 contains the supplementary crystallographic data for this paper. These data can be obtained free of charge via https://www.ccdc.cam.ac.uk/structures/ (accessed on 25 August 2024) (or from the CCDC, Cambridge, UK).

### 3.4. Synthesis of the Complexes

Complexes **2, 3** and **5** [Au(SIPr)(C≡C–aryl)] were prepared following a general synthetic method [59] with a slight modification. Briefly, the starting material [Au(SIPr)Cl] (0.08 mmol), sodium acetate (0.24 mmol) and the corresponding alkyne (0.19 mmol) were deposited in a vial and suspended in EtOH (2 mL). The reaction mixture was stirred at room temperature for 16 h and protected from light. After that time, the solvent was removed under vacuum. The solid residue was dissolved in CH_2_Cl_2_ (2 mL), and the solution was filtered through Kieselgur. The filtrate was concentrated under vacuum, and the product was precipitated by adding *n*-hexane (3 mL). The solid was collected, washed three times with diethyl ether and dried under vacuum.

*[Au(SIPr)(C≡C*–*(2-phenanthrenyl)]* (**2**). Yield 58%. Anal. Calcd for C_43_H_47_N_2_Au: C, 65.47; H, 6.02; N, 3.55. Found: C, 65.41; H, 6.27; N, 3.15. Ir (KBr, cm^−1^); 2962(ν_C–H_), 2104(ν_C≡C_), 1490(ν_CCarom_), 1475(ν_CCarom_). ^1^H NMR (300 MHz, DMSO-*d*_6_) δ 7.44 (dt, *J* = 7.4, 5.7 Hz, 2H), 7.34 (d, *J* = 7.7 Hz, 4H), 7.18–7.00 (m, 5H), 4.07 (s, 4H), 3.11 (sept, *J* = 6.8 Hz, 4H), 1.36 (d, *J* = 6.8 Hz, 12H), 1.32 (d, *J* = 6.9 Hz, 12H). ^13^C NMR (75 MHz, DMSO-d_6_) δ 208.47, 146.56, 146.09, 138.26, 134.51, 131.39, 131.34, 129.57, 129.46, 129.23, 128.55, 127.81, 126.86, 126.67, 124.34, 122.90, 122.61, 122.06, 101.31, 53.78, 28.26, 28.25, 25.01, 23.70.

*[Au(SIPr)(C≡C*−*(3-chlorophenyl]* (**3**). Yield 32 %. Anal. Calcd. For C_35_H_42_N_2_AuCl: C, 58.13; H, 5.87; N, 3.87. Found: C, 58.14; H, 5.90; N, 3.69. Ir (KBr, cm^−1^): 2960(ν_C–H_), 2114(ν_C≡C_), 1588(ν_CCarom_), 1491(ν_CCarom_), 1275(ν_C–N_), 684(ν_C–Cl_). ^1^H NMR (300 MHz, DMSO-d_6_) δ 7.46 (dd, *J* = 8.5, 6.9 Hz, 2H), 7.34 (d, *J* = 7.7 Hz, 4H), 7.16–7.12 (m, 2H), 7.07 (d, *J* = 1.5 Hz, 1H), 7.04–6.99 (m, 1H), 4.08 (s, 4H), 3.11 (sept, *J* = 6.8 Hz, 4H), 1.35 (d, *J* = 12.0 Hz, 12H), 1.32 (d, *J* = 6.9 Hz, 12H). ^13^C NMR (75 MHz, DMSO-d_6_) δ 208.19, 146.51, 134.70, 134.45, 132.61, 130.54, 129.82, 129.78, 129.57, 127.79, 125.98, 124.34, 102.07, 53.76, 28.21, 25.04, 23.65.

*[Au(SIPr)(C≡C*−*(2-pyridyl]* (**4**). The synthesis of this compound required the previous deprotonation of the alkyne. The ethynylpyridine (27 mg, 0.262 mmol) was dissolved in 5 mL of a solution of 0.11 M of sodium methoxide in MeOH and maintained for 30 min with stirring. [Au(SIPr)Cl] (80 mg, 0.129 mmol) was added to the solution, and the mixture was reacted for 24 h. Following work-up was analogous to that described in the general procedure. Yield 79 %. Anal. Calcd. For C_34_H_42_N_3_Au: C, 59.21; H, 6.15; N, 6.09. Found: C, 58.92; H, 6.16; N, 5.86. Ir (KBr, cm^−1^): 2960(ν_C–H_), 2118(ν_C≡C_), 1583(ν_CNpyridine_), 1490(ν_CCarom_), 1460(ν_−Carom_), 1275(ν_C–N_). ^1^H NMR (300 MHz, DMSO-d_6_) δ 8.29–8.25 (m, 1H), 7.55–7.48 (m, 1H), 7.48–7.41 (m, 2H), 7.34 (d, *J* = 7.6 Hz, 4H), 7.09–7.03 (m, 2H), 4.09 (s, 4H), 3.11 (sept, *J* = 6.8 Hz, 4H), 1.36 (d, *J* = 6.8 Hz, 12H), 1.32 (d, *J* = 6.8 Hz, 12H). ^13^C NMR (75 MHz, DMSO-d_6_) δ 208.20, 149.15, 146.50, 144.58, 135.77, 134.38, 133.21, 129.59, 126.23, 124.31, 120.94, 103.69, 53.74, 28.21, 25.05, 23.66.

*[Au(SIPr)(C≡C*−*(4-bromophenyl]* (**5**)**.** Yield 29 %. Anal. Calcd. For C_35_H_42_N_2_AuBr: C, 54.76; H, 5.53; N, 3.65. Found: C, 54.81; H, 5.57; N, 3.53. Ir (KBr, cm^−1^): 2961(ν_C–H_), 2112(ν_C≡C_), 1489(ν_CCarom_), 1458(ν_CCarom_), 1272(ν_C–N_), 619(ν_C–Br_). ^1^H NMR (300 MHz, DMSO-d_6_) δ 7.45 (dd, *J* = 8.5, 6.9 Hz, 2H), 7.33 (d, *J* = 7.7 Hz, 4H), 7.31–7.26 (m, 2H), 7.03–6.96 (m, 2H), 4.07 (s, 4H), 3.10 (sept, *J* = 6.9 Hz, 4H), 1.35 (d, *J* = 6.9 Hz, 12H), 1.31 (d, *J* = 6.9 Hz, 12H). ^13^C NMR (75 MHz, DMSO-d_6_) δ 208.28, 146.50, 134.45, 134.27, 133.10, 130.91, 129.55, 125.08, 124.33, 118.84, 102.41, 53.74, 28.20, 25.03, 23.65.

## 4. Conclusions

The reaction of [Au(SIPr)Cl] with different arylalkynes in the presence of a base yielded complexes **1**–**5,** which were characterized and whose cyclic voltammetry was explored both under argon and under CO_2_ atmosphere. In the cyclic voltammetry measurements under argon atmosphere, complexes **2**–**4** display irreversible reduction at values ranging between −2.79 and −2.91 V referenced to the ferrocenium/ferrocene couple. Complex **1** does not show this reduction, and this fact can be understood by comparing the energies of the LUMOs of the complexes. The energy of the LUMO of complex **1** is higher than the energy of the LUMOs of the other compounds, and consequently complex **1** is expected to experience a reduction at a lower potential than that which should appear outside of the measurement window of the experiment. The DFT calculations show that the LUMO is mainly contributed to by the atomic orbitals of atoms of the aryl–C≡C fragments with little participation from the atomic gold orbitals, and the reduction takes place mainly in the aryl–C≡C fragments. The cyclic voltammetry measurements performed under carbon dioxide atmosphere showed an increase in the faradaic current of the reduction wave of these complexes, indicating that there is activity towards the CO_2_.

## Figures and Tables

**Table 1:** Crystallographic data for 2.

Empirical Formula	C_43_H_47_AuN_2_	Formula Weight	788.79
temperature/K	240	crystal system	orthorhombic
space group	*P*2_1_2_1_2_1_		
*a*/Å	13.1684 (4)	*α*/deg	90
*b*/Å	16.4356 (5)	*β*/deg	90
*c*/Å	38.3544 (12)	*γ*/deg	90
volume/Å^3^	8301.1 (4)	Z	8
*ρ*_calc_ g/cm^3^	1.262	*μ*/mm^−1^	6.856
*F* (000)	3184.0	radiation	Cu K_α_ (*λ* = 1.54178)
2Θ range for data collection/deg	4.07 to 72.23	index ranges	−15 ≤ *h* ≤ 16−20 ≤ *k* ≤ 20−47 ≤ *l* ≤ 45
reflections collected	16064	goodness-of-fit on *F*^2^	1.035
final R indexes [*I* ≥ 2*σ* (*I*)]	*R*_1_ = 0.0288	final [all data]	*R*_1_ = 0.0328
	w*R*_2_ = 0.0700		w*R*_2_ = 0.0730
largest diff. peak/hole/e Å^−3^	0.732/−0.584	CCDC number	2,363,526

**Table 2:** Selected bond lengths (Å), angles (deg) and torsion angles (deg) for 2.

	XRD	DFT		XRD	DFT
Au2–C60	2.036 (6)	2.045	Au2–C59	2.003 (7)	2.008
Au1–C17	2.033 (6)	2.045	Au1–C16	1.983 (6)	2.008
N3–C60	1.311 (8)	1.336	N3–C61	1.471 (8)	1.479
N2–C17	1.329 (8)	1.337	N2–C19	1.469 (8)	1.479
N4–C60	1.331 (8)	1.336	N4–C62	1.463 (8)	1.479
N1–C17	1.320 (8)	1.337	N1–C18	1.470 (8)	1.479
C61–C62	1.527 (10)	1.542	C14–C15	1.433 (9)	1.426
C16–C15	1.207 (9)	1.228	C19–C18	1.502 (10)	1.539
C57–C58	1.442 (9)	1.426	C58–C59	1.192 (9)	1.228
C59–Au2–C60	176.1 (3)	179.1	C16–Au1–C17	173.8 (3)	179.0
C60–N3–C61	113.4 (5)	113.1	C17–N2–C19	111.7 (5)	112.8
N3–C60–Au2	126.7 (4)	125.0	N3–C60–N4	108.7 (5)	109.1
N4–C60–Au2	124.5 (4)	126.0	C60–N4–C62	112.7 (5)	113.0
N2–C17–Au1	126.5 (5)	125.7	N1–C17–Au1	123.5 (5)	125.3
N1–C17–N2	109.5 (5)	109.0	C17–N1–C18	112.7 (5)	112.8
C58–C59–Au2	171.1 (6)	178.6	N3–C61–C62	101.6 (5)	102.3
C15–C16–Au1	168.2 (6)	178.3	N2–C19–C18	103.5 (5)	102.2
N4–C62–C61	102.2 (5)	102.4	N1–C18–C19	102.5 (5)	102.2
C60–N3–C75–C76	91.7 (8)	87.7	C60–N3–C75–C79	88.5 (8)	91.6
C60–N4–C63–C68	84.2 (8)	87.2	C60–N4–C63–C64	97.1 (8)	92.1
C17–N2–C20–C25	96.6 (8)	95.7	C17–N2–C20–C21	82.3 (9)	83.9
C17–N1–C32–C33	82.2 (9)	96.9	C17–N1–C32–C37	98.8 (8)	82.6

**Table 3:** Cyclic voltammetry data for compounds **1**–**5** in MeCN and Bu_4_PF_6_ supporting electrolyte, using the redox system ferrocenium/ferrocene as internal reference *^a^*.

Compound	$E_{p}^{\mathrm{red}}$(V) Ar *^b^*	*i*_Ar_(*μ*A) *^c^*	$E_{p}^{\mathrm{red}}$(V) CO_2_*^d^*	*i*_CO2_(*μ*A) *^e^*	${\mathrm{Ratio}}^{\;f}\;\frac{i_{CO2}}{i_{Ar}}$
**1**			−2.99	−92.74	
**2**	−2.87(ir)	−38.36	−2.88	−95.58	2.49
**3**	−2.90(ir)	−29.11	−3.04	−56.50	1.94
**4**	−2.91(ir)	−46.51	−1.40/−3.00	−35.5/−82.49	1.77
**5**	−2.79(ir)	−26.46/	−1.37/−3.04	−18.49/−68.94	2.61

*^a^* The reduction potential mean value observed for ferrocenium/ferrocene (Fc^+^/Fc), used as an internal calibrant under the employed experimental conditions, was E° = 0.476 V vs. the AgCl/Ag (3 M KCl) electrode. *^b^* Cathodic peaks observed under argon. *^c^* Maximum registered cathodic current under argon. *^d^* Cathodic peaks observed under CO_2_. *^e^* Maximum registered cathodic current under CO_2_. *^f^* Ratio between the faradaic currents observed under CO_2_ (*i*_CO2_) and under argon (*i*_Ar_).

## Data Availability

Data are contained within the article and Supplementary Materials.

## Supplementary Materials

The following supporting information can be downloaded at: https://www.mdpi.com/1420-3049/29/17/4081/s1, Figures S1–S3: intermolecular interactions in the crystal structure; Figures S4–S8: Cyclic voltammograms of complexes **1**–**5**; Figures S9–S12: energy levels and frontier molecular orbitals of complexes **1**, **3**–**5**; Table S1: Composition of the molecular orbitals for complexes **1**–**5**; Figures S13–S16; ^1^H and ^13^C NMR spectra for complexes **2**–**5**.
