# Supplementary material for: Synthesis and Electrochemical Study of Gold(I) Carbene Complexes
Source: Molecules. 2024 Aug 28;29(17):4081. doi: 10.3390/molecules29174081 (PMC11487389; doi:10.3390/molecules29174081)
Supplement: Supplementary file 1 [file molecules-29-04081-s001.zip › molecules-3092416-supplementary.pdf]

Supporting Information

Synthesis and Electrochemical Study of Gold(I) Carbene Complexes

Andrea Rodríguez-Rubio, Álvaro Yuste, Tomás Torroba, Gabriel García-Herbosa and José V. Cuevas-Vicario *

Departamento de Química, Facultad de Ciencias, Universidad de Burgos, 09001 Burgos, Spain
* Correspondence: jvcv@ubu.es

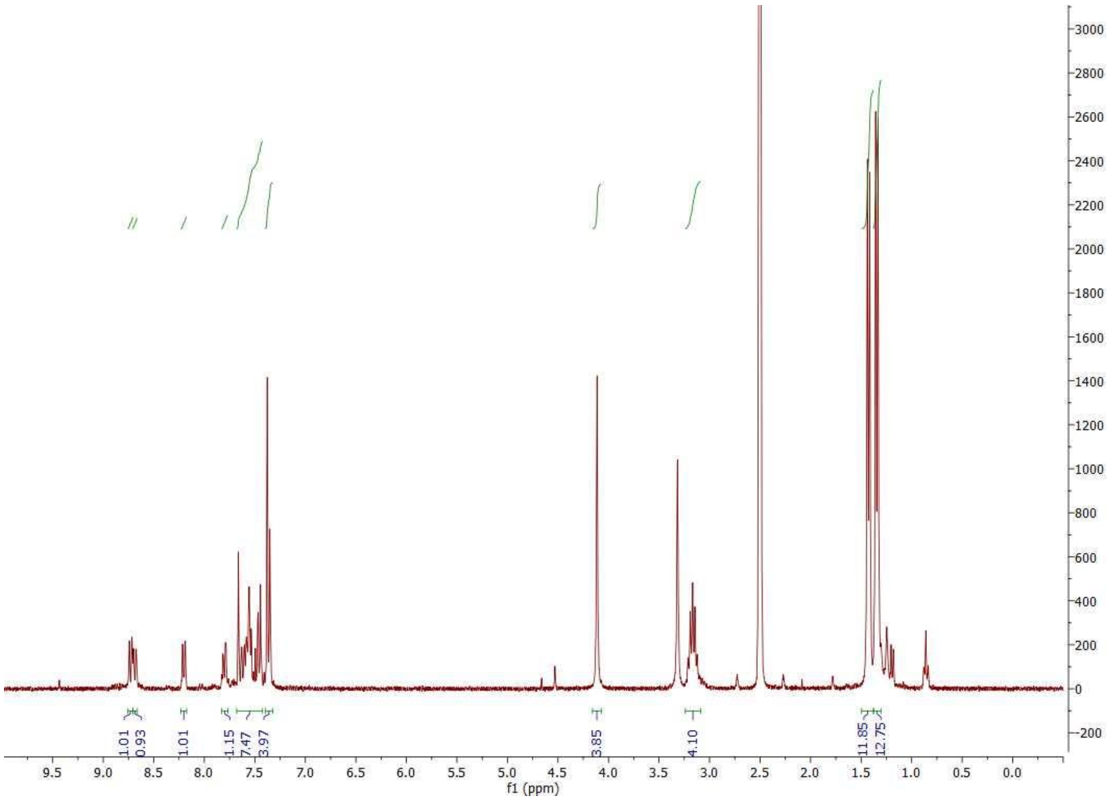

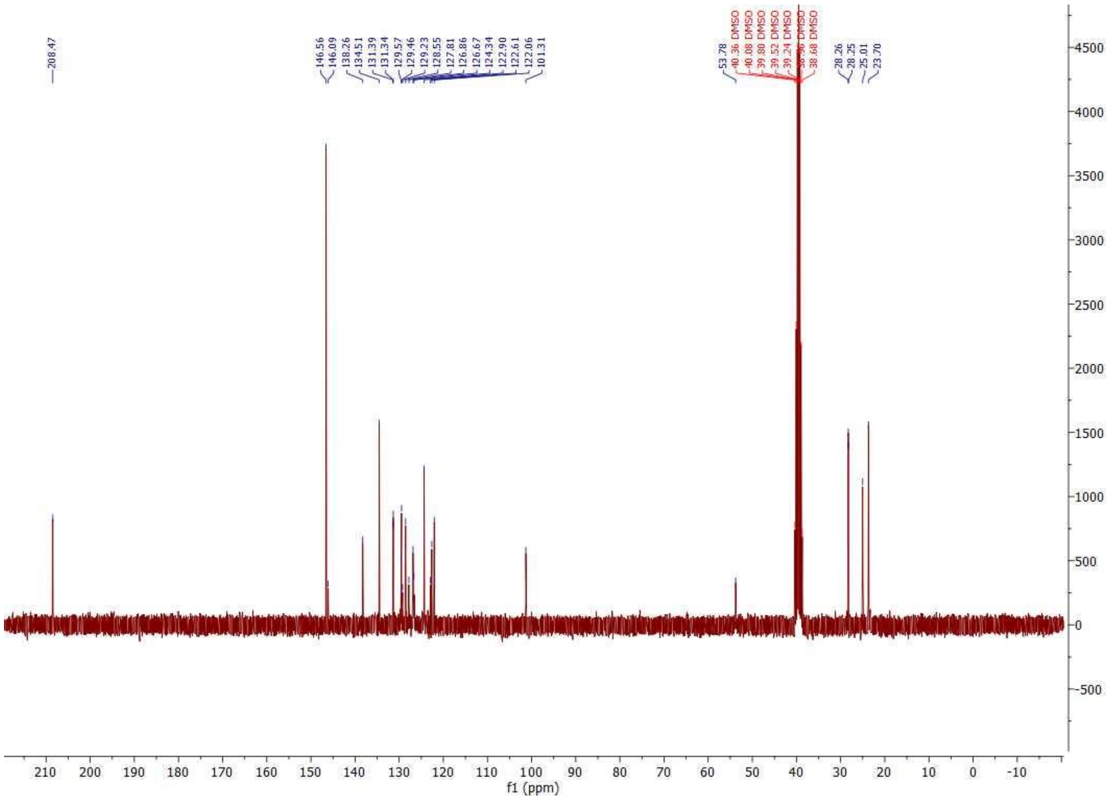

Figure S1. ^{1}H NMR (up) and ^{13}C NMR spectra of compound **2**.

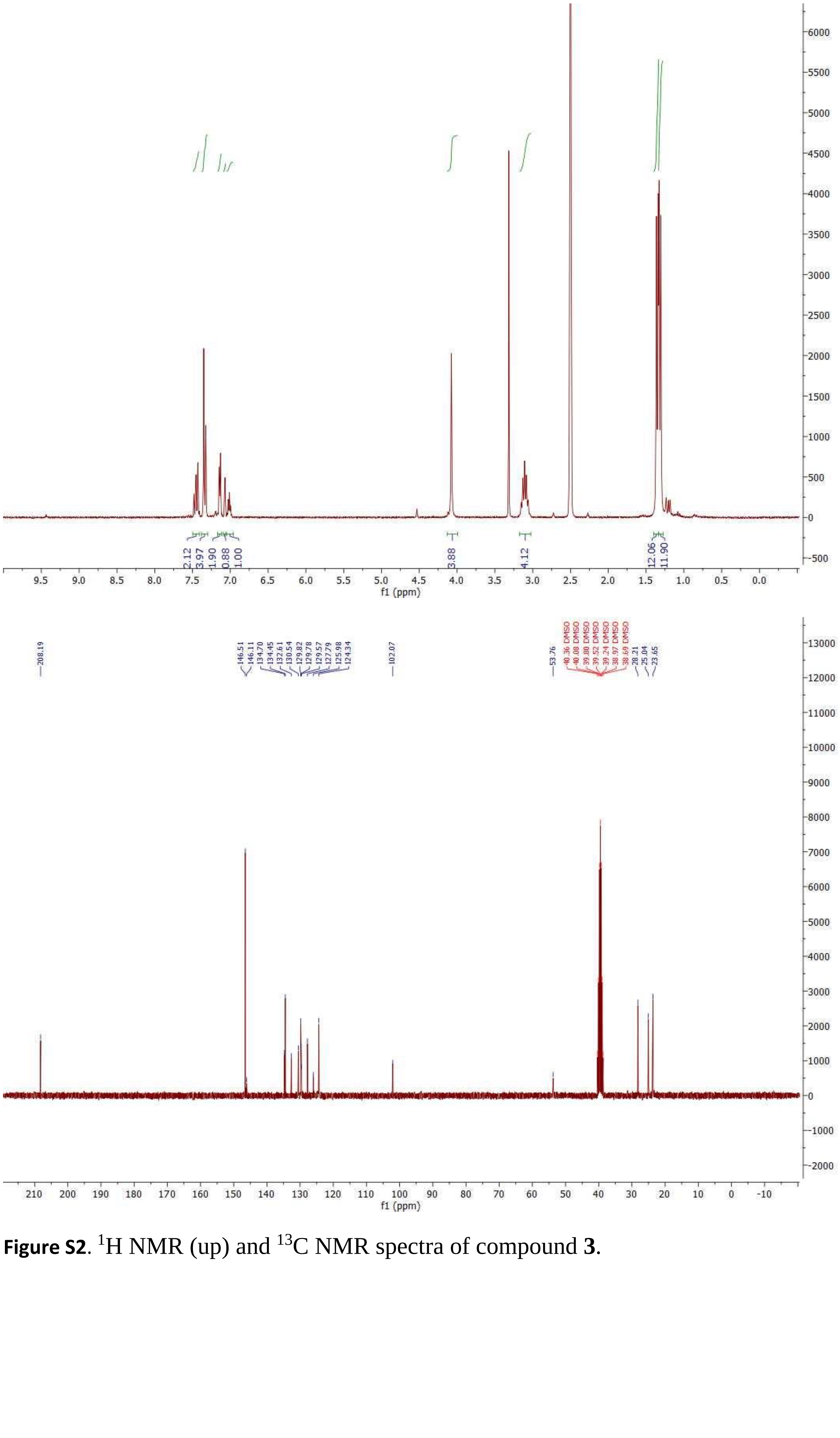

Figure S2. ^{1}H NMR (up) and ^{13}C NMR spectra of compound **3**.

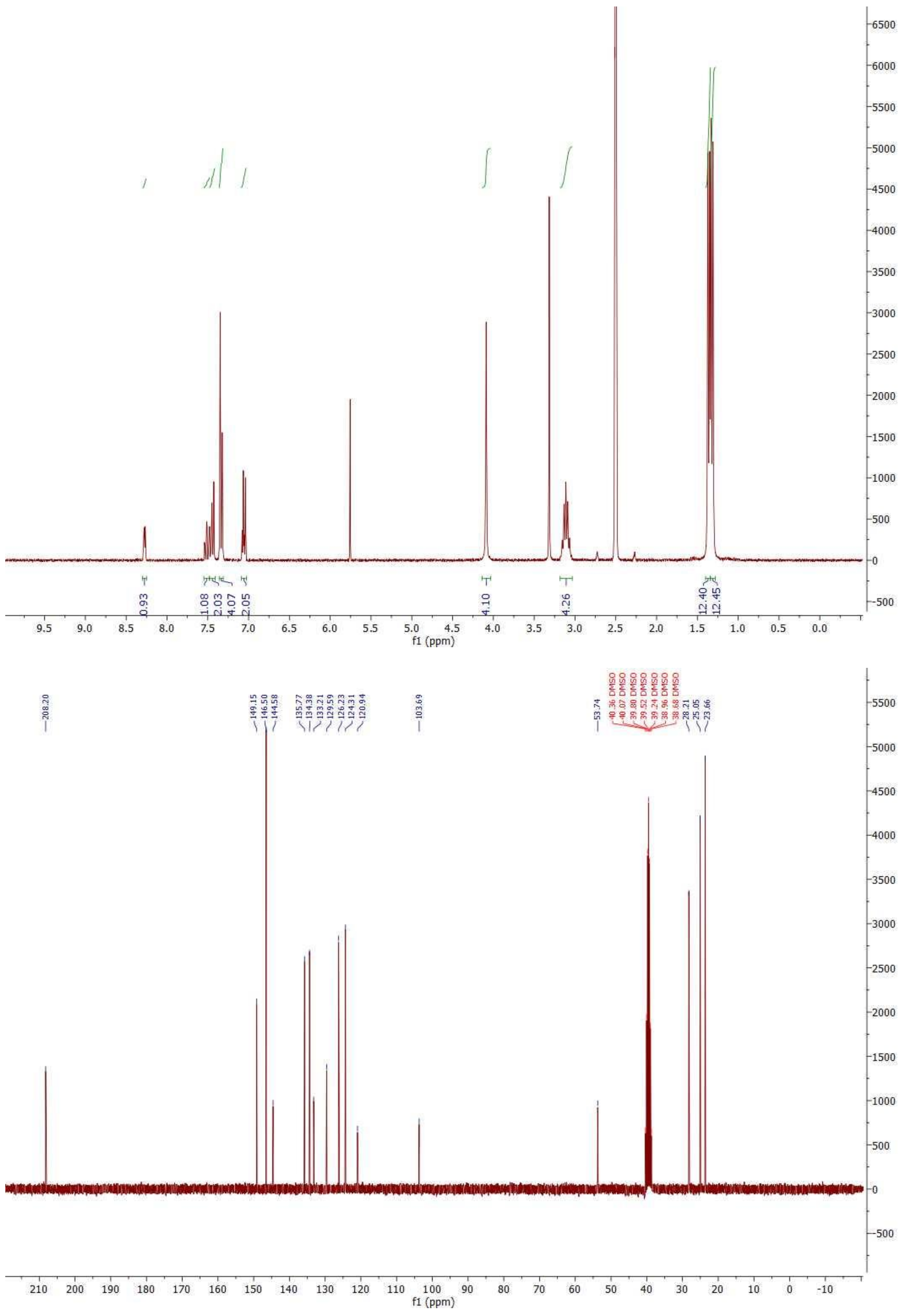

Figure S3. ^{1}H NMR (up) and ^{13}C NMR spectra of compound **4**.

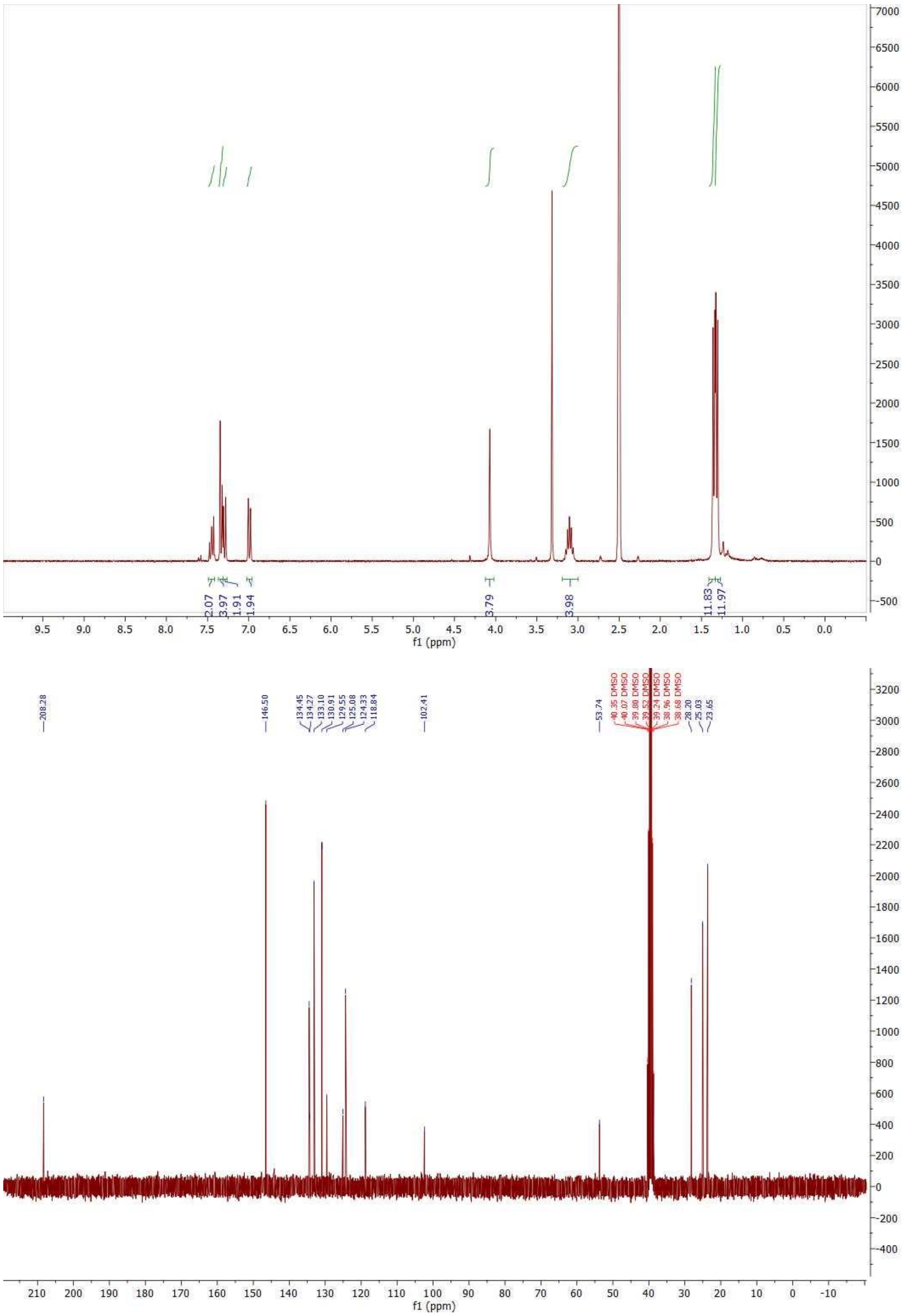

Figure S4. ^{1}H NMR (up) and ^{13}C NMR spectra of compound **5**.

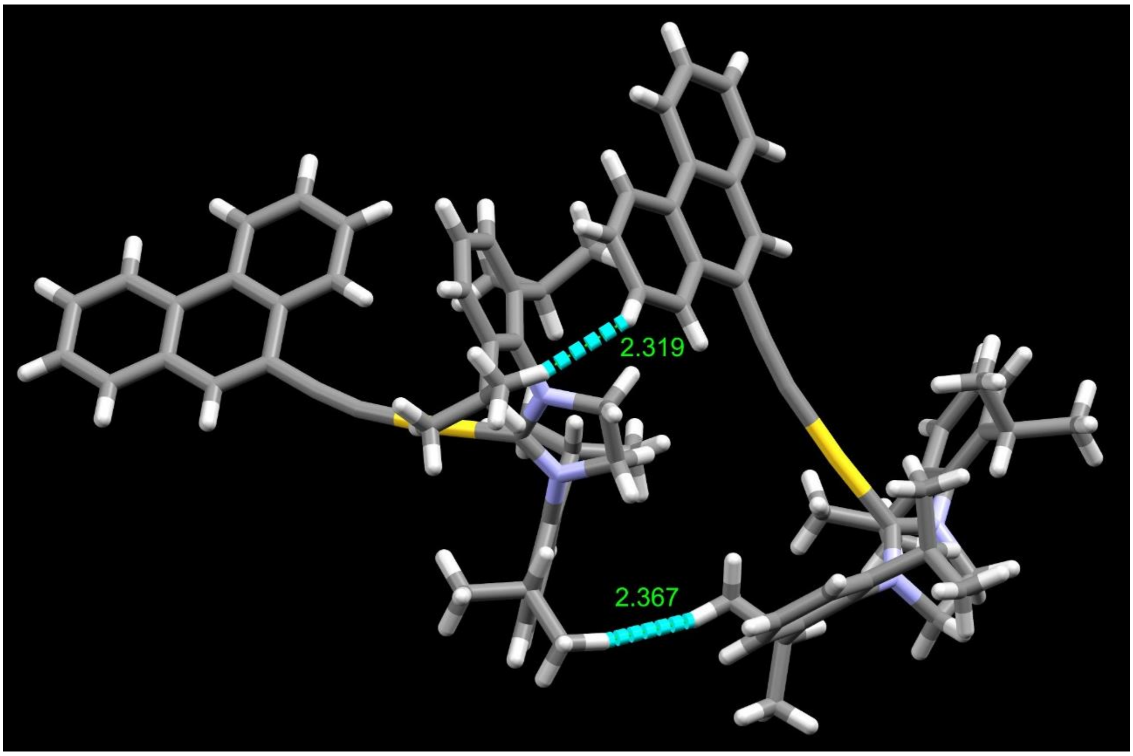

Figure S5. CH···HC interactions in two fragments of the asymmetric unit.

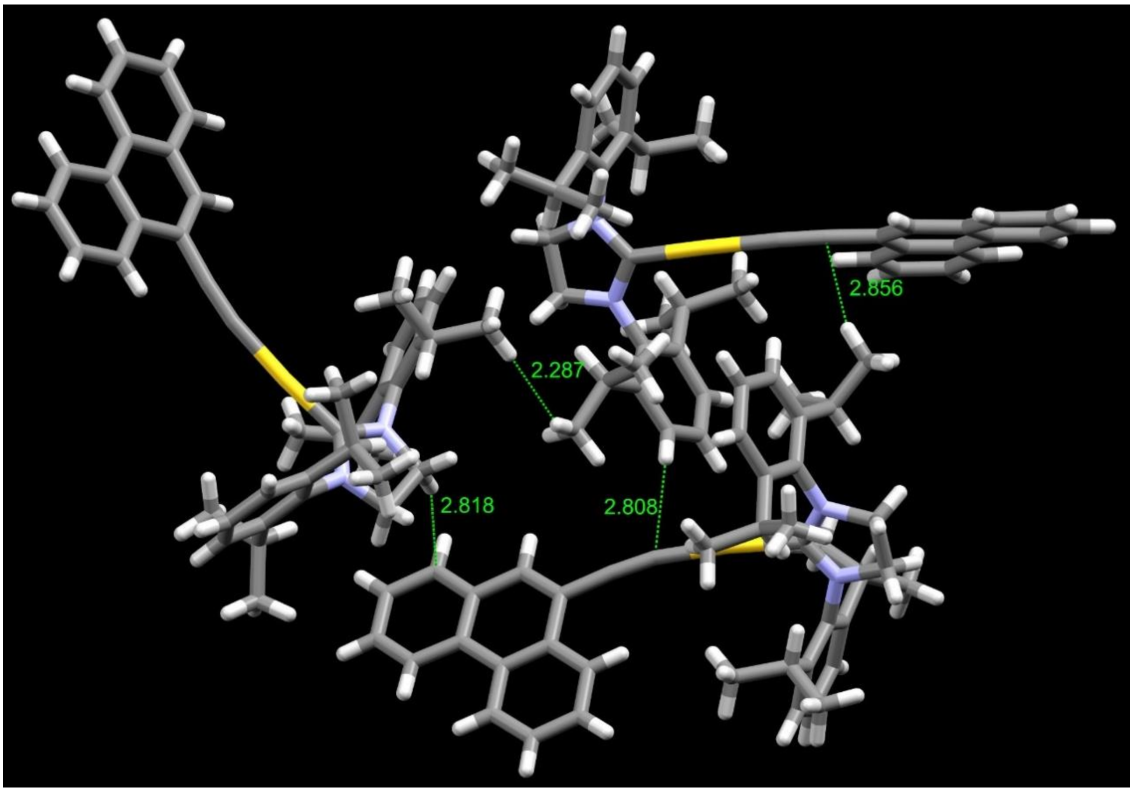

Figure S6. Intermolecular CH···HC and C-H···π interactions in two fragments of the asymmetric unit.

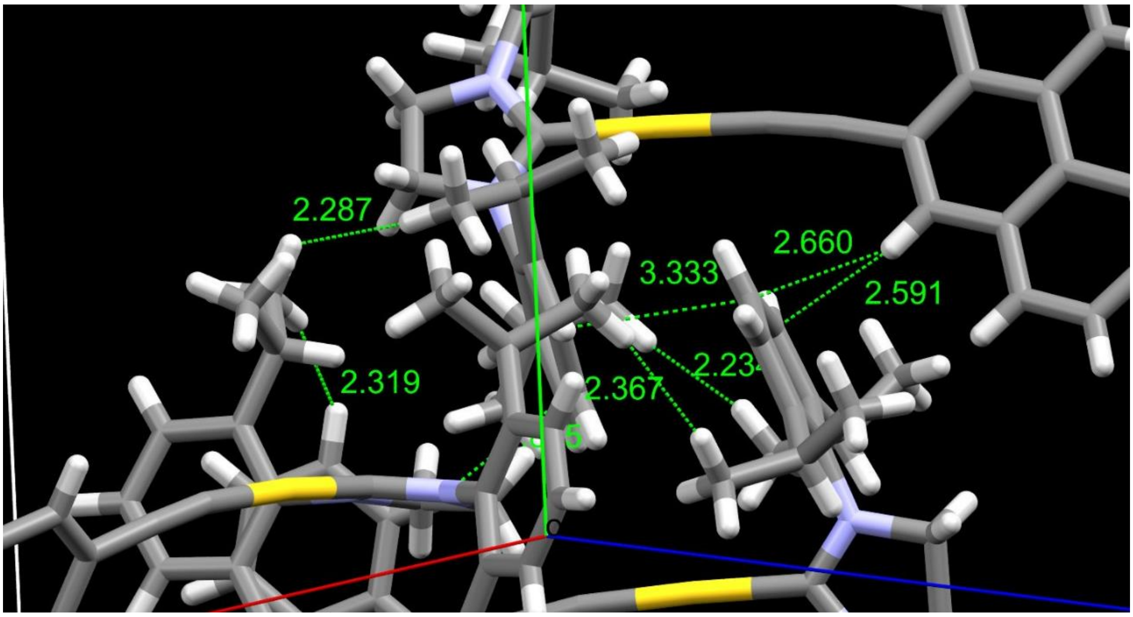
2.287
2.660
3.333
2.591
2.319
2.367

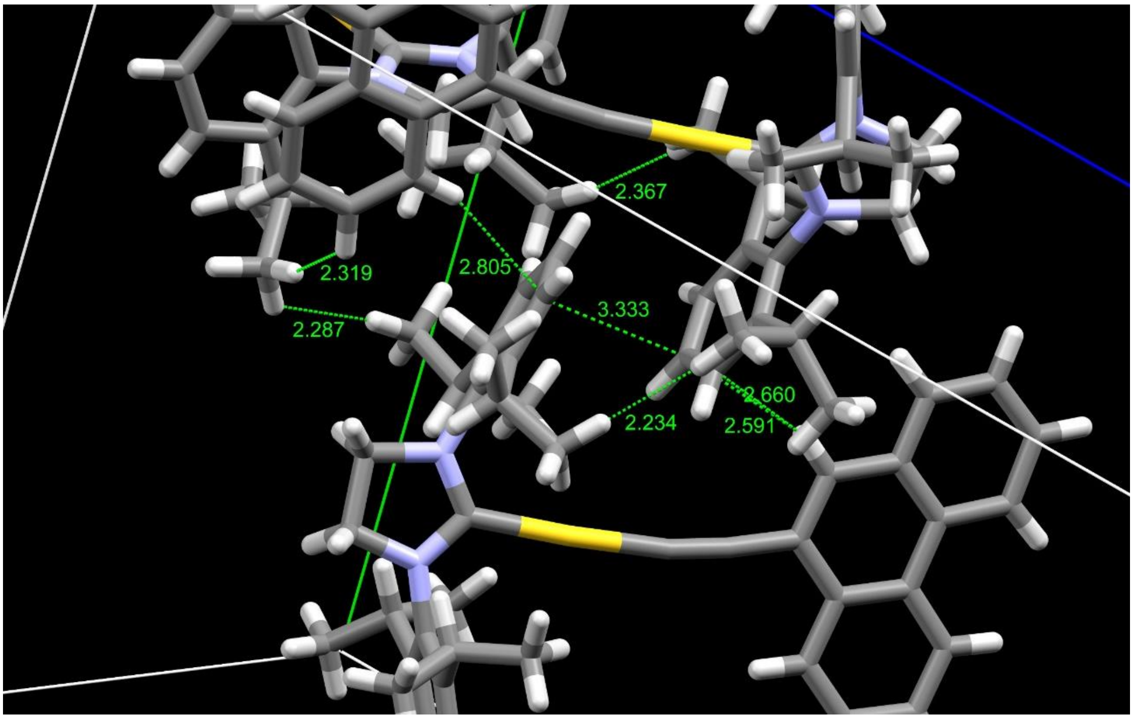
2.367
2.319
2.805
3.333
2.287
2.660
2.234
2.591

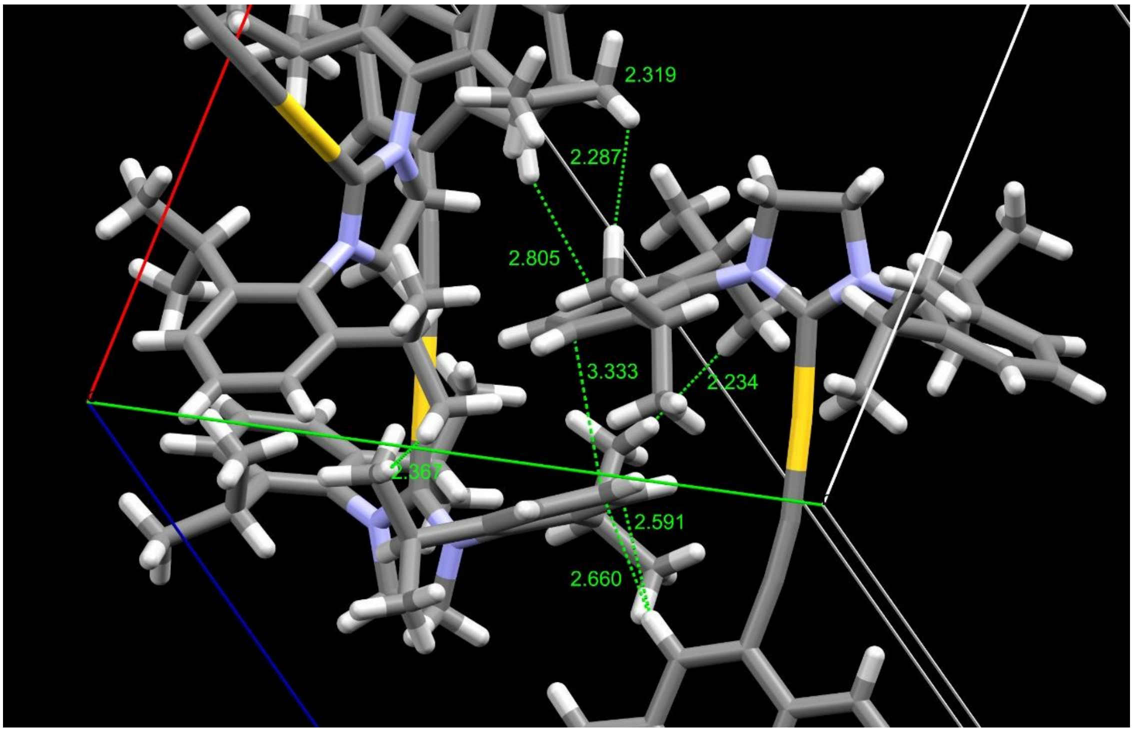

Figure S7. Three orientations of the intermolecular CH···HC and C-H···π interactions.

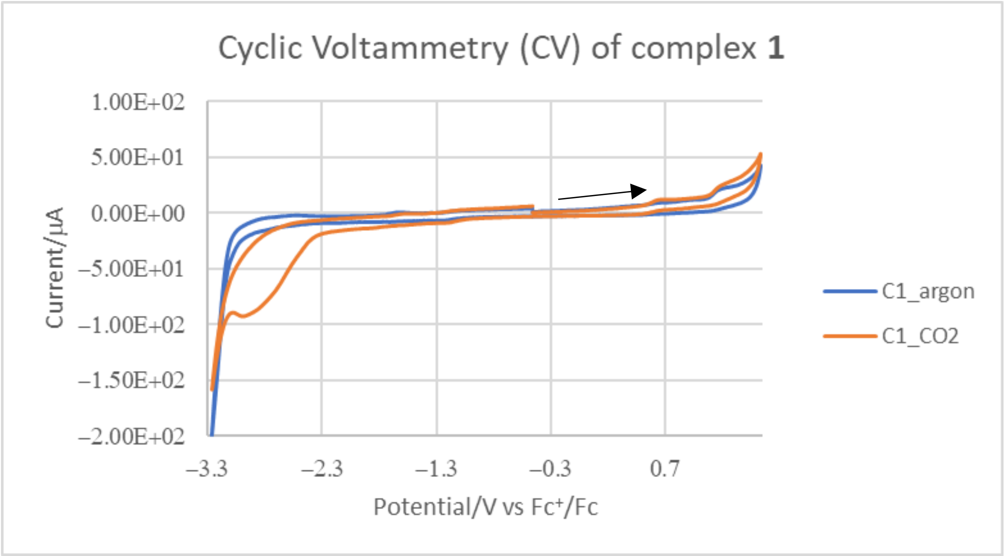

Figure S8. Cyclic voltammogram of complex **1** ($5\cdot10^{-4}$ M, glassy carbon working electrode dish 3.0 mm diameter, dry MeCN, 0.1 M Bu_4NPF_6, scan rate 100 $V\cdot s^{-1}$) recorded under argon atmosphere (blue line) and CO_2 atmosphere (orange line). The arrow indicates the scan direction.

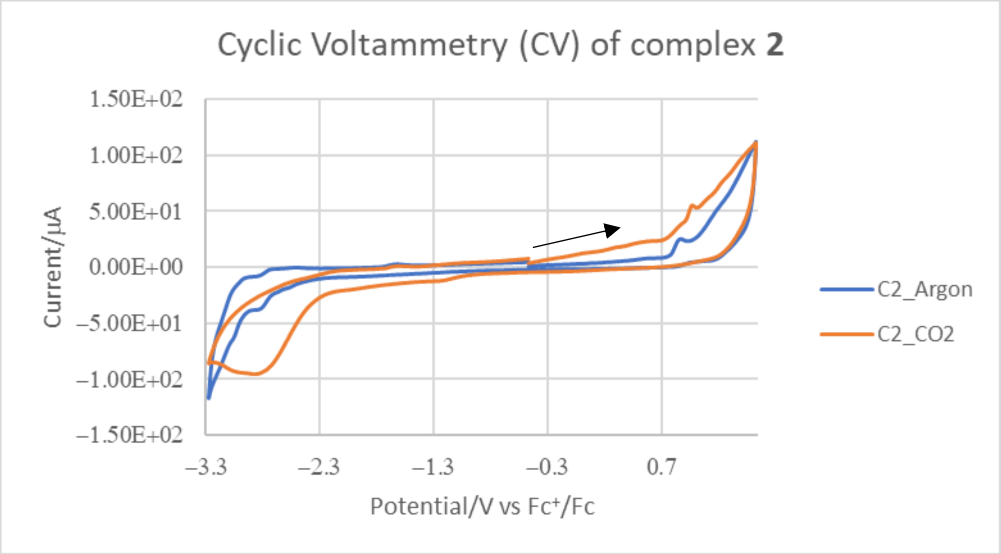

Figure S9. Cyclic voltammogram of complex **2** (5·10^{-4} M, glassy carbon working electrode dish 3.0 mm diameter, dry MeCN, 0.1 M Bu_4NPF_6, scan rate 100 V·s^{-1}) recorded under argon atmosphere (blue line) and CO_2 atmosphere (orange line). The arrow indicates the scan direction

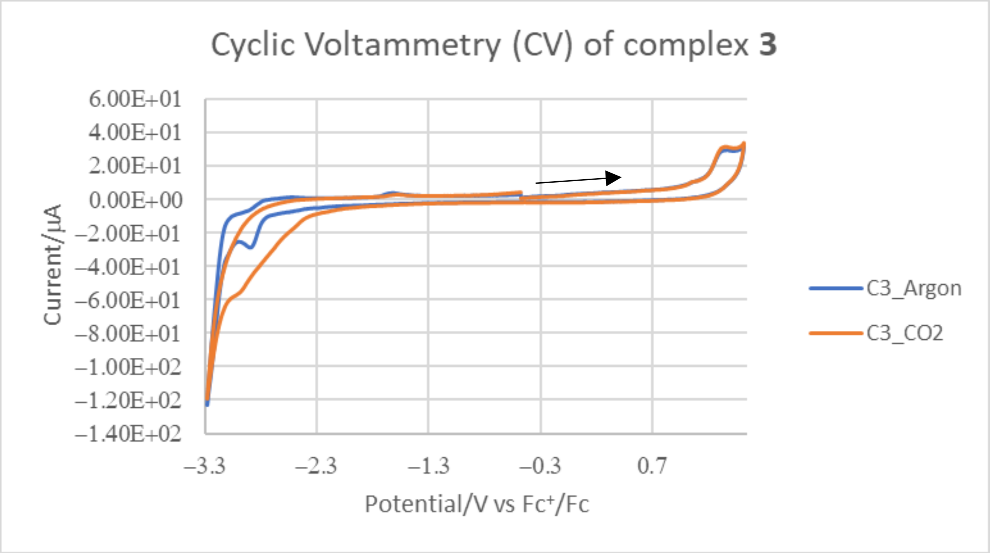

Figure S10. Cyclic voltammogram of complex **3** (5·10^{-4} M, glassy carbon working electrode dish 3.0 mm diameter, dry MeCN, 0.1 M Bu_4NPF_6, scan rate 100 V·s^{-1}) recorded under argon atmosphere (blue line) and CO_2 atmosphere (orange line). The arrow indicates the scan direction

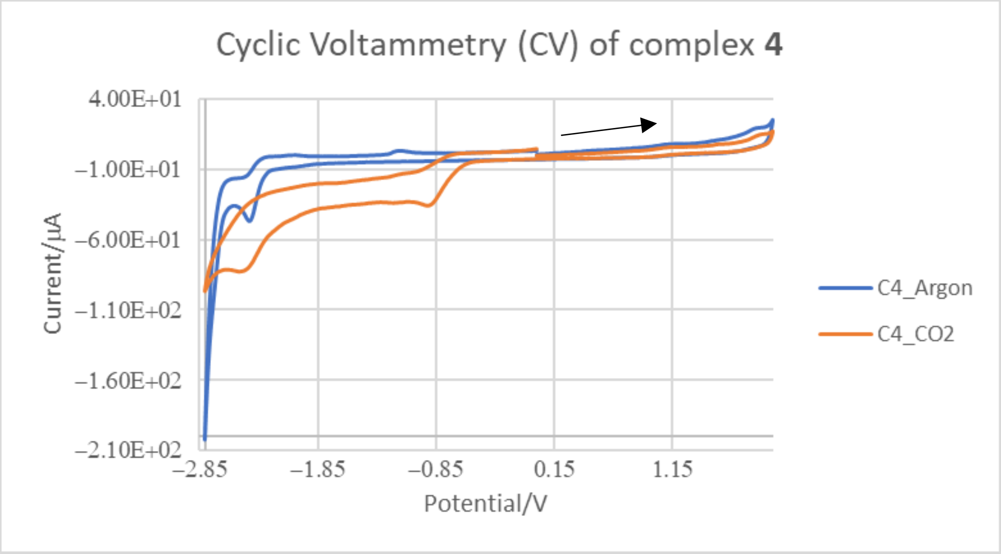

Figure S11. Cyclic voltammogram of complex **4** ($5 \cdot 10^{-4}$ M, glassy carbon working electrode dish 3.0 mm diameter, dry MeCN, 0.1 M Bu_4NPF_6, scan rate 100 $V \cdot s^{-1}$) recorded under argon atmosphere (blue line) and CO_2 atmosphere (orange line). The arrow indicates the scan direction

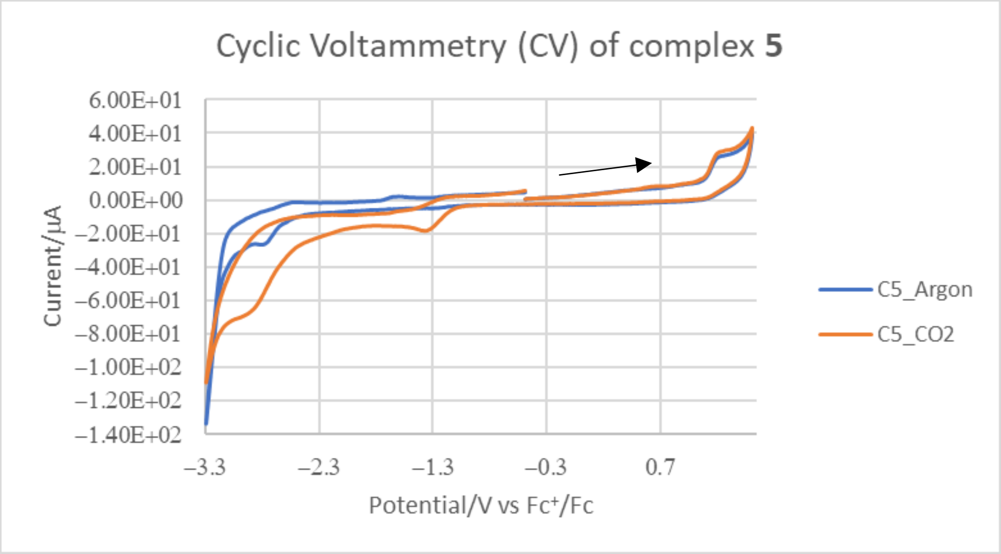

Figure S12. Cyclic voltammogram of complex **5** ($5 \cdot 10^{-4}$ M, glassy carbon working electrode dish 3.0 mm diameter, dry MeCN, 0.1 M Bu_4NPF_6, scan rate 100 $V \cdot s^{-1}$) recorded under argon atmosphere (blue line) and CO_2 atmosphere (orange line). The arrow indicates the scan direction

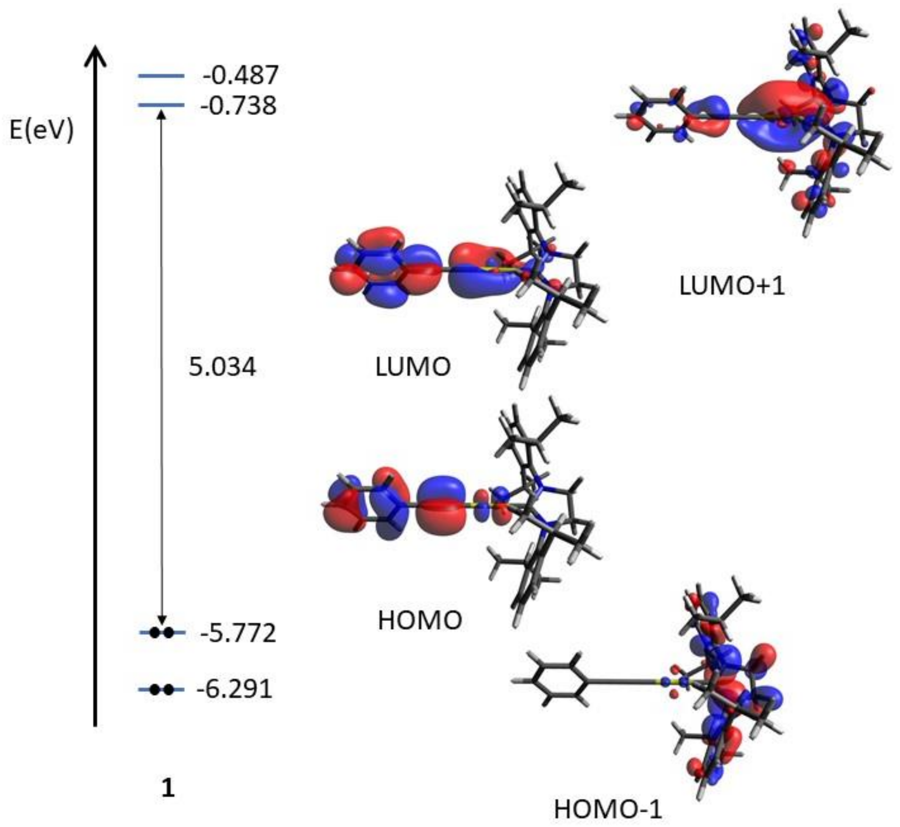

Figure S13. Energy levels and isosurface contour plots (0.03 au) for complex **1**.

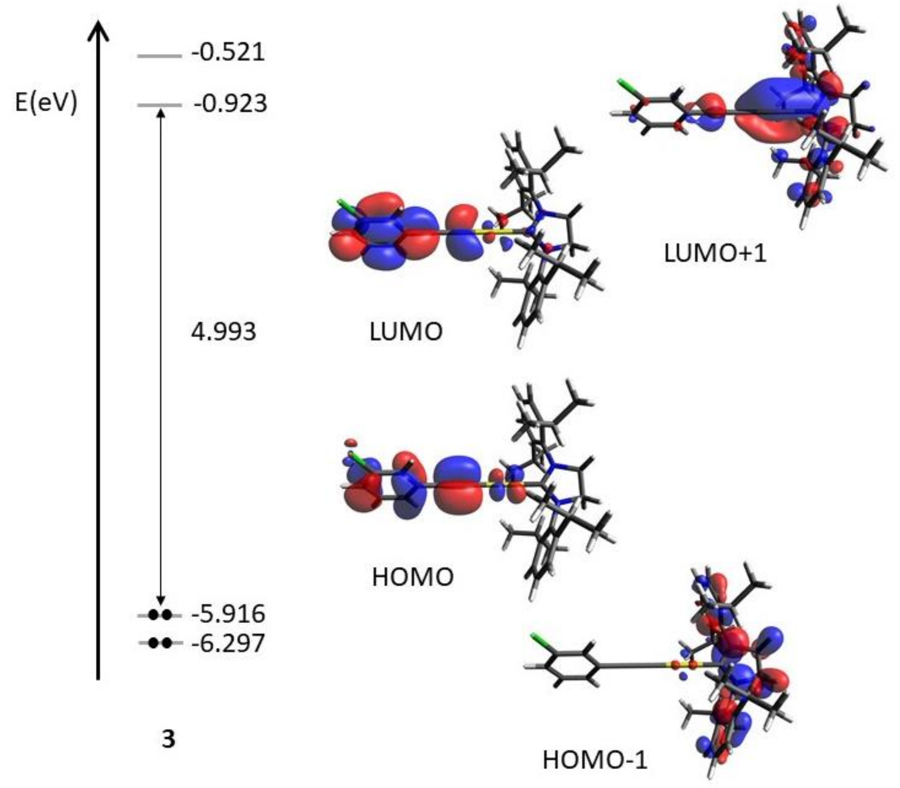

Figure S14. Energy levels and isosurface contour plots (0.03 au) for complex **3**.

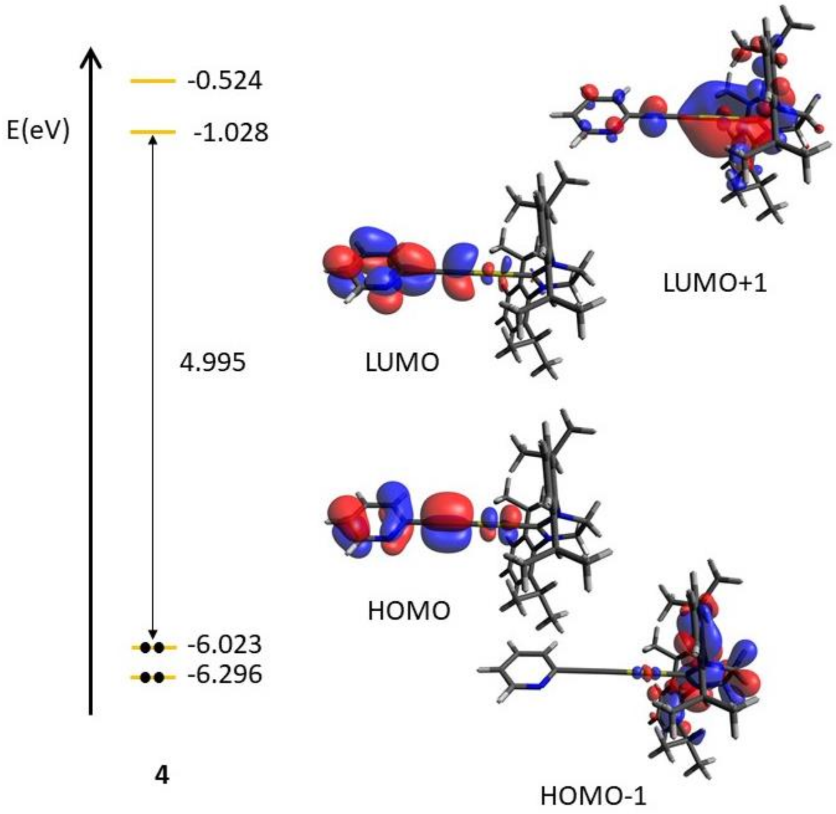

Figure S15. Energy levels and isosurface contour plots (0.03 au) for complex **4**.

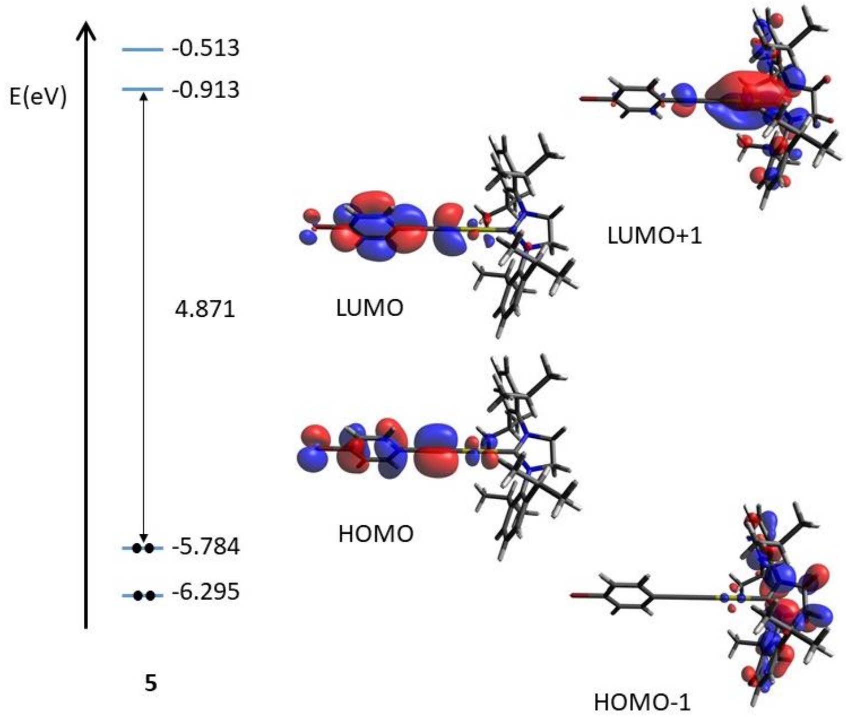

Figure S16. Energy levels and isosurface contour plots (0.03 au) for complex **5**.

Table S1. Composition of the molecular orbitals.

	HOMO-1	HOMO	LUMO	LUMO+1
**1**				
Au	2,33	14,08	13,71	15,95
Carbene	97,51	2,66	13,32	69,74
C≡C-Aryl	0,16	83,26	72,96	14,31
**2**				
Au	0,17	7,41	5,00	0,06
Carbene	0,04	1,56	2,65	0,03
C≡C-Aryl	99,79	91,03	92,35	99,90
**3**				
Au	2,15	14,75	10,42	16,47
Carbene	97,71	2,75	7,17	70,92
C≡C-Aryl	0,14	82,50	82,41	12,61
**4**				
Au	2,14	15,65	8,87	16,47
Carbene	97,72	2,95	5,42	70,70
C≡C-Aryl	0,14	81,40	85,71	12,83
**5**				
Au	2,20	11,89	10,05	16,67
Carbene	97,65	2,24	6,60	72,65
C≡C-Aryl	0,15	85,87	83,35	10,68
